# Human papillomavirus partial L2 gene variants in the Barrett’s metaplasia-dysplasia-adenocarcinoma sequence

**DOI:** 10.1128/mra.00551-25

**Published:** 2025-10-07

**Authors:** Kishen Rajendra, Aliakbar Khabiri, Shanmugarajah Rajendra, Mohammad Rabiei

**Affiliations:** 1 Ingham Institute for Applied Medical Research, Gastro-Intestinal Viral Oncology Group, Sydney, Australia; 2 School of Medicine, University of Southampton 7423, Southampton, United Kingdom; 3 Institute for Applied Ecology, University of Canberra 2234, Canberra, Australia; 4 Southwestern Sydney Clinical School, University of New South Wales, Sydney, Australia; 5 Department of Gastroenterology and Hepatology, Bankstown-Lidcombe Hospital, Sydney, Australia; Katholieke Universiteit Leuven, Leuven, Belgium

**Keywords:** human papillomavirus (HPV), oesophageal adenocarcinoma (OAC), phylogenetic study

## Abstract

High-risk human papillomavirus (HPV) genotypes 16 and 18 are associated with Barrett’s dysplasia and esophageal adenocarcinoma. We sequenced 33 HPV partial L2 genes from Australian esophageal specimens. Phylogenetic analysis showed 32 were HPV-16 and one was HPV-18, underscoring the predominance and importance of HPV-16.

## ANNOUNCEMENT

Oncogenic viruses are responsible for a substantial percentage of global cancer cases (1). Human papillomavirus (HPV), a key player, is linked to approximately 30% of virus-related cancers (2). HPV is a small, double-stranded DNA virus that belongs to the *Papillomaviridae* family with an ~8 kb genome divided into early (E), late (L), and long control (LCR) regions; types are classified based on <90% L1 gene nucleotide similarity, subtypes on 90–98%, and variants on >98% similarity (3). The L2 gene was selected in this study because its partial conservation made it a suitable target for broad primers, while its higher divergence provided better variant discrimination than L1 (4).

In esophageal adenocarcinoma (OAC), studies have indicated that high-risk HPV is associated with 25% of malignancies (5, 6). In this study, we report the partial L2 genes of HPV strains detected from OAC cases from Bankstown-Lidcombe Hospital in Sydney, Australia, and compare them phylogenetically to previously reported strains. Viral genome sequences are essential for understanding the genetic diversity, evolution, and epidemiology of HPV.

From 2012 to 2014, we collected 33 biopsies from patients with Barrett’s metaplasia–dysplasia–adenocarcinoma (median age 46, range 42–79). Samples were homogenized, and viral DNA was extracted using the DNeasy Blood & Tissue Kit (Qiagen, USA). DNA quality and concentration were assessed with a NanoDrop spectrophotometer. The partial L2 region was amplified by PCR with type-specific primers (Table 1), visualized on agarose gels, and purified (Qiagen, USA). Amplicons (90–100 bp) were sequenced bidirectionally by the Sanger method at the Ramaciotti Centre for Genomics (UNSW, Sydney). Consensus sequences were compared with GenBank references, showing >99% similarity, and the most closely related strains were used for phylogenetic analysis. A maximum likelihood tree was constructed in MEGA X to determine evolutionary relationships.

**TABLE 1:** PCR conditions for amplification of HPV L2 gene regions

Component	HPV-16 (HQ644299)	HPV-18 (GQ180792)	Thermal cycling conditions
Forward primer (5’→3’)	AAC CGA AAT CGG TTG AAC CG	GGA GTA ACC GAA AAC GGT	**Initial denaturation:** 95°C, 2 min **35 cycles:** - Denaturation: 95°C, 30 s - Annealing: 57°C, 30 s - Extension: 72°C, 1 min **Final extension:** 72°C
Reverse primer (5’→3’)	TGA TGT GTA TGT AGA CAC AGA C	CAT ACA TGC ATA CAC AAA AGC	
Target region	Partial L2 gene	Partial L2 gene	
Reference sequence	HQ644299	GQ180792	

**Fig 1:**
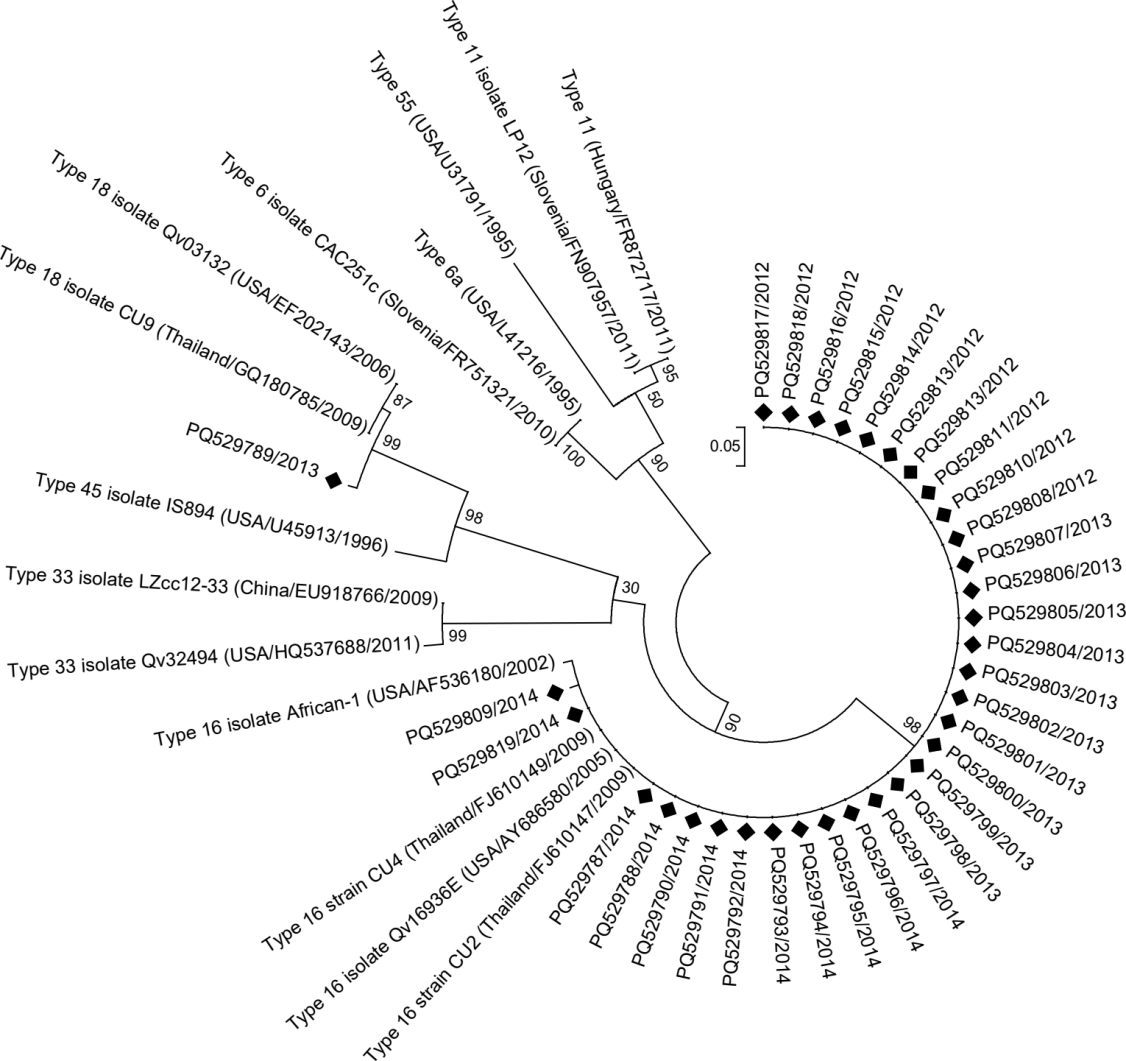
Phylogenetic tree based on the partial L2 gene sequences of HPV. Raw sequence reads from forward and reverse primers were assembled and trimmed to generate consensus sequences for each sample using BioEdit software. Consensus sequences were compared with published sequences in the NCBI database using BLASTn. Multiple sequence alignment was performed using Clustal Omega (https://www.ebi.ac.uk/Tools/msa/clustalo/) with available sequences from GenBank. The alignment included 47 nucleotide sequences, with codon positions 1st+2nd+3rd+Noncoding. All positions containing gaps and missing data were eliminated, resulting in a final data set of 81 positions. The phylogenetic tree was constructed using the Maximum Likelihood method in MEGA X (7) with 1,000 bootstrap replications, applying the maximum composite likelihood model. Evolutionary analyses were conducted in MEGA X. All HPV16 variants were highly similar, with pairwise sequence similarity ranging from 99.79% to 100%, mainly because of the sequence length.

Sequences showed >99% similarity with GenBank references. One sequence clustered with HPV-18 strains CU9 (Thailand) and Qv03132 (USA), while 32 clustered with HPV-16 strains CU2/CU4 (Thailand) and African-1 (USA) (Fig. 1).

The predominance of HPV-16 among the samples aligns with global epidemiological trends, highlighting its significant role in oncogenesis (8). The clustering patterns observed could inform future public health strategies and vaccination programs. Additionally, the identification of HPV-18, although less frequent, emphasizes the need for comprehensive screening and monitoring to address all high-risk HPV types effectively.

## Data Availability

The partial L2 gene sequences have been deposited in GenBank under accession numbers PQ529787, PQ529788, PQ529789, PQ529790, PQ529791, PQ529792, PQ529793, PQ529794, PQ529795, PQ529796, PQ529797, PQ529798, PQ529799, PQ529800, PQ529801, PQ529802, PQ529803, PQ529804, PQ529805, PQ529806, PQ529807, PQ529808, PQ529809, PQ529810, PQ529811, PQ529812, PQ529813, PQ529814, PQ529815, PQ529816, PQ529817, PQ529818, and PQ529819, and PRJNA1207125 (SRA).
